# Full genome sequence analysis and putative host-shifting of *Milk vetch dwarf virus* infecting tobacco (*Nicotiana tabacum*) in China

**DOI:** 10.1186/s12985-019-1129-5

**Published:** 2019-03-27

**Authors:** Ali Kamran, Han Hou, Yi Xie, Cunxiao Zhao, Xiaomin Wei, Chaoqun Zhang, Xiangwen Yu, Fenglong Wang, Jinguang Yang

**Affiliations:** 1 Key Laboratory of Tobacco Pest Monitoring Controlling & Integrated Management, Tobacco Research Institute of Chinese Academy of Agricultural Sciences, Qingdao, 266101 China; 2 Graduate School of Chinese Academy of Agricultural Sciences, Beijing, 100081 China; 3 Qingyang Tobacco Company, Gansu Tobacco Cooperation, Xifeng, 745000 China; 4 Jiangxi Tobacco Science Institute, Nanchang, 330025 China; 5 Sichuan Tobacco Science Institute, Chengdu, 610000 China

**Keywords:** Nanovirus, Rolling circle amplification, Full genome sequence, *Milk vetch dwarf virus*, MDV, *N. tabacum*, Recombination, Host-shifting

## Abstract

**Background:**

Tobacco production in China has been affected by plant viruses with Milk vetch dwarf virus (MDV) as a recent invader posing serious concern. According to most of the studies, MDV mainly infects hosts from Fabaceae family but in our previous study we reported its infection in tobacco plant (*Nicotiana tabacum* L.) in Shandong province.

**Findings:**

In current study (2016–2017), tobacco plants (*Nicotiana tabacum*) with severe stunting, yellowing and axillary bunch of new leaves were observed in Zhengning, Gansu province. Isolate GSZN yielded into eight genomic circular single-stranded DNA components while no alphasatellite DNA was obtained. High percent identity of this isolate was recorded in overall nucleotide and amino acid assembly with reported MDV isolates worldwide. Phylogenetic analysis fetched into a separate sub-clade comprising of new isolate along with other tobacco infecting isolates of MDV. While recombination was predicted in DNA-C encoding Clink protein and DNA-U1, which may attribute towards the potential host-shifting phenomenon and ability of this virus to expand its host range.

**Conclusion:**

To our knowledge this is the first full genome annotation of a Nanovirus, infecting tobacco in natural field conditions, also this is the first extended analysis on host-shifting behavior of MDV.

**Electronic supplementary material:**

The online version of this article (10.1186/s12985-019-1129-5) contains supplementary material, which is available to authorized users.

## Main text

Tobacco is an important economic crop worldwide, with half of its farming in China; world’s largest producer with 1,259,549 ha of cultivated area, producing 2,806,770 t annually [1]. Viral diseases have been reported as a potential threat to tobacco production in China, where 25 plant viruses are considered to be involved [2–4]. Among them, *Milk vetch dwarf virus* (MDV) has caused a serious damage to legume crops throughout the Japan as well as in central and south China, since its first detection in 1968 [5–8]. Despite the preference of legumes, recently a report was published regarding MDV infection causing severe stunting and crinkling in tobacco (*N. tabacum*) from an eastern province of China [4].

MDV is a member of genus Nanovirus in family Nanovirideae and transmit by aphid vector in a persistent manner. The genome is circular single-stranded DNA (cssDNA), comprising of at least eight genomic (~ 1 kb each) and occasionally three or four alphasattelite DNAs that encapsidate separately as isometric particles of 18 nm giving multipartite genomic assembly to MDV. Each genomic DNA encode a distinct protein i.e., DNA-C, DNA-N, DNA-M, DNA-R, and DNA-S encodes; cell-cycle link protein (Clink), nuclear-shuttle protein (NSP), movement protein (MP) master replication protein (M-Rep) and capsid protein (CP), respectively [7, 9, 10], while the functions for DNA-U1, DNA-U2 and DNA-U4 are unknown [11].

Yang et al. [4], presented preliminary research, including analysis of only two genomic DNAs i.e. DNA-R and DNA-S sequences that provoke further study for this potential and emerging constraint to tobacco production as well as host-shifting of MDV in natural field conditions. In current study we have identified, cloned and sequenced all eight genomic cssDNAs sequences of GSZN isolate of MDV, infecting tobacco plants in Zhengning city, Gansu province in northwest of China. Due to the economic importance of the crop and persistence of the disease in two consecutive years, we further investigated the etiological agent to adopt appropriate measures accordingly, that will surely help farmers to avoid the particular viral attack that might be turn into epidemic if not addressed at this stage.

## Materials and methods

**Fig. 1:**
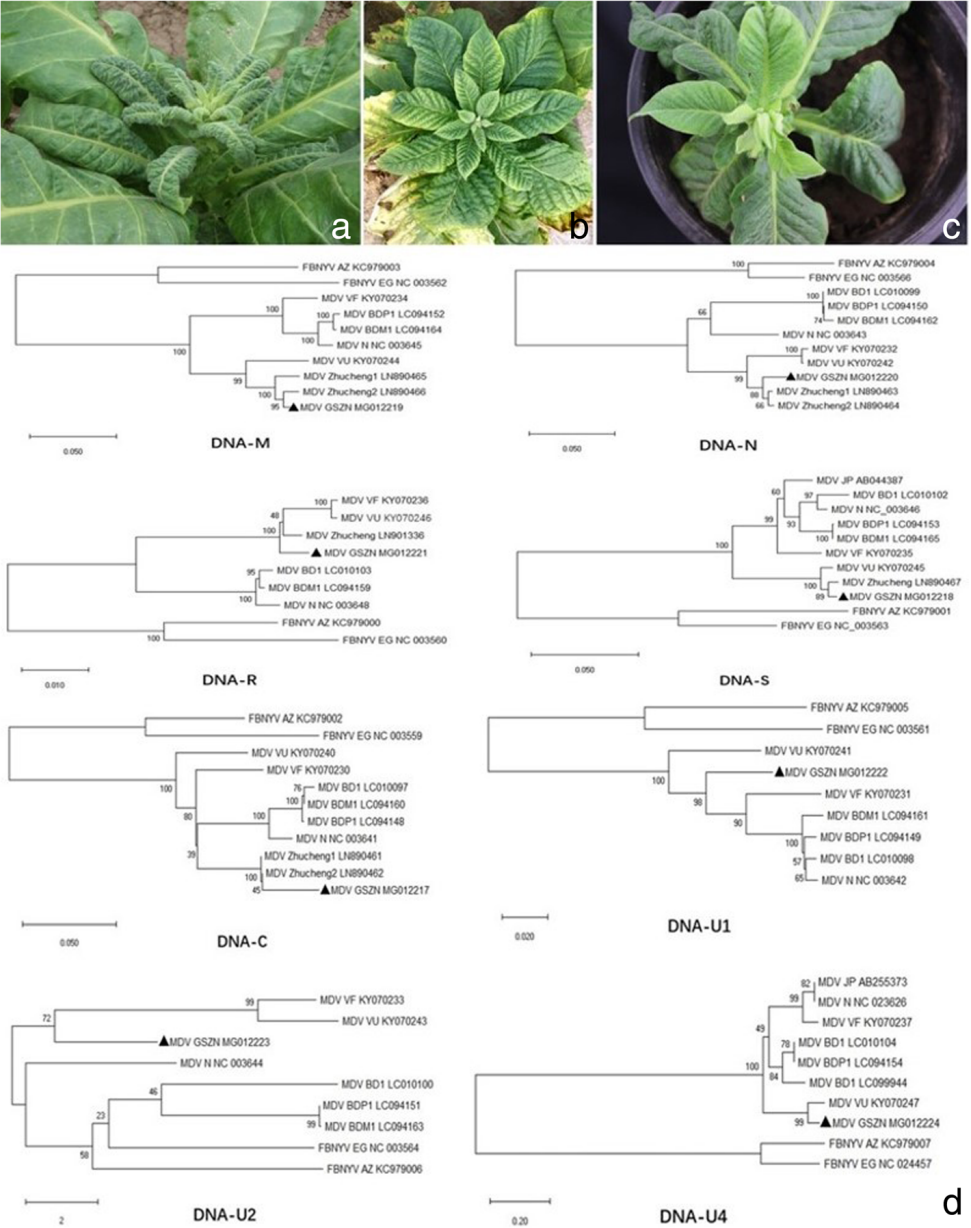
Symptoms observed on tobacco plants during field survey: **a** axillary bunch of new leaves (**b**) stunting and interveinal yellowing (**c**) symptoms expression by aphid transmission of MDV (**d**) Phylogenetic analysis based on the nucleotide sequences of all genomic components of reported MDV isolates and GSZN isolate (reported herein) along with another Nanovirus; FBNYV. The tree was constructed by the neighbor- joining method using the MEGAX program. Bootstrap analysis was applied using 1000 replicates

During the spring, 2016-2017, tobacco plants (N. tabacum) were observed showing severe stunting, interveinal yellowing, leaf crinkling and axillary bunch of new leaves, in Zhengning city with disease incidence of 4.1% (Fig. 1a and b). On the bases of the characteristic symptoms of Nanovirus infection observed in the field from eight different locations, total DNA was extracted from 49 samples, using Easy Pure Plant Genomic DNA Kit (TransGen Biotech, Beijing, China) for downstream experiments to confirm the potential etiological agent of the disease. Owing to the single-stranded circular genome of Nanoviruses, rolling circle amplification (RCA) [12] was carried out using TempliPhi™ Kit (GE Healthcare, Fisher Scientific, USA) according to manufacturer’s protocol. Viral DNA and pUC19 (Fisher Scientific, USA) digested with the same restriction enzymes i.e. *Xba*I, *Sma*I, *Sal*I, *Pst*I, *Bam*HI, *Eco*RI, *Hind*III, and *Sac*I (Additional file 1: Figure S1) and resulted fragments were recovered by Easy pure gel DNA extraction kit (TransGen Biotech, Beijing, China). The extracted fragments were then dephosphorylated and ligated using T4-DNA ligase (Fisher Scientific, USA) followed by transformation into *Escherichia coli*. Universal primer pair: M13F:5′-TGTAAAACGACGGCCAGT-3, M13R:5′-CAGGAAACAGCTATGACC-3 was used for colony identification and the fragments of interest were sequenced from all randomly selected clones at Sangon Biotech Co. Ltd. (Shanghai, China), through Sanger sequencing. However, no alphasatellite DNA was amplified using three sets of specific primers i.e. C2F:5’-ACAGATGTAGAGAGAGAAACAT-3’, C2R: 5’-AAGAAGGTTCATTTAATTGTGT-3’, C3F: 5’-TAATGACCGCGTTCAGTACG-3’ C3R:5’- TCCCAAGAGTAGCGTCTGAG-3’ C10F:5’- CATGAGAGAGTGAATCACGA-3’, and C10R: 5’-TTAATTACGCAGTAATTGAG-3’ for DNAC2, DNAC3, and DNAC10, respectively. All the obtained sequences were analyzed and submitted to GenBank under the accession numbers: MG012217- MG012224. Phylogenetic analysis was performed using MegaX software using neighbour joining method [13], whereas recombination events were predicted using RDP4.95 software [14]. Insect transmission experiment was performed using *Myzus persicae* in greenhouse followed by PCR assay using primer pair: MDVCP-F: 5′-CTGGTGCGGGGCTTAGTATTACCCCCGCA-3′, and MDVCP-R:5′- CGGGATCAAATGACGTCACAAGGTCATAT-3′ (amplicon size: 1003 bp) to confirm the MDV infection, 30 days post-inoculation.

## Results and discussion

Two Samples showed presence of Nanovirus infection through RCA. All eight genomic components were retrieved from selected isolate GSZN by direct sequencing of RCA product. BLAST showed similarity with the corresponding sequences of MDV. Whereas, using DNAMAN software, high level of percent nucleotide (nt) (99.20–79.33%) as well as amino acid (aa) identity (100–82.67%) except DNA-U2 (73.60%) was estimated between GSZN isolate and most of the corresponding sequences available in GenBank, while an overall low percent identity was depicted in DNA-U4 (Table 1). The trend of low percent similarity in the above mentioned genomic components, makes the isolate GSZN more vulnerable of possible recombination. Moreover, the phylogenetic analysis performed using MegaX software, depicted that isolate GSZN is placed among the tobacco infecting isolates i.e. Zhucheng 1 and 2 in all the available sequences of these two isolates that strongly justify the grouping of MDV on the basis of host plant infectivity (Fig. 1d) Furthermore, leaf crinkling, stunting and axillary bunch of new leaves were observed after 30 days of insect transmission experiment (Fig. 1c) followed by confirmation of MDV in 12 out of 14 tested plants through PCR assay depositing 1003 bp product on 1% agarose gel electrophoresis (Additional file 2: Figure S2). Same results were also confirmed by loop-mediated isothermal amplification (LAMP) [15].

**Table 1:** Percent identity of genomic DANs of isolate GSZN and corresponding sequences in GenBank

	Zhucheng1	Zhucheng2	VU^b^	VF^b^	BDP1^b^	BDM1^b^	BD1^b^	N^b^	JP^b^
**DNA-U1**	**–**	**–**	**88.89%** ^a^	**87.90%**	**92.82%**	**92.72%**	**92.92%**	**88.64%**	**–**
	**–**	**–**	*90.67%* ^*a*^	*–*	*82.67%*	*84.00%*	*84.87%*	*85.33%*	**–**
**DNA-U2**	**–**	**–**	**93.89%**	**82.95%**	**83.92%**	**84.12%**	**81.68%**	**86.17%**	**–**
	**–**	**–**	*92.00%*	*73.60%*	*90.40%*	*90.40%*	*88.00%*	*89.60%*	**–**
**DNA-U4**	**–**	**–**	**91.12%**	**80.10%**	**75.70%**	**–**	**73.75%**	**80.18%**	**80.18%**
	**–**	**–**	*96.30%*	*83.33%*	*90.74%*	*–*	*89.81%*	*75.61%*	*75.61%*
**DNA-C**	**96.38%**	**96.38%**	**87.99%**	**88.90%**	**84.76%**	**84.76%**	**84.27%**	**89.44%**	**–**
	*100%*	*100%*	*90.53%*	*92.31%*	*92.31%*	*92.31%*	*91.12%*	*92.31%*	–
**DNA-S**	**98.11%**	**–**	**98.50%**	**91.48%**	**88.02%**	**88.10%**	**87.52%**	**92.28%**	**91.98%**
	*98.84%*	*–*	*98.84%*	*94.77%*	*93.60%*	*93.68%*	*93.02%*	*94.19%*	*93.60%*
**DNA-R**	**99.20%**	**–**	**98.70%**	**98.70%**	**–**	**91.88%**	**91.78%**	**95.31%**	**–**
	*98.60%*	*–*	*98.95%*	*98.95%*	*–*	*96.50%*	*96.15%*	*95.80%*	–
**DNA-N**	**97.60%**	**97.81%**	**92.62%**	**92.52%**	**82.85%**	**82.45%**	**82.85%**	**88.25%**	–
	*97.39%*	*97.39%*	*95.42%*	*94.77%*	*96.08%*	*94.77%*	*96.08%*	*95.42%*	–
**DNA-M**	**96.66%**	**97.98%**	**92.02%**	**84.26%**	**79.33%**	**79.53%**	**79.92%**	**83.15%**	–
	*97.32%*	*100%*	*99.11%*	*94.64%*	*92.86%*	*92.86%*	*93.75%*	*93.75%*	–

^a^ = amino acid identity: italic, nucleotide identity: bold; ^b^ = name of the isolate of MDV

**Fig. 2:**
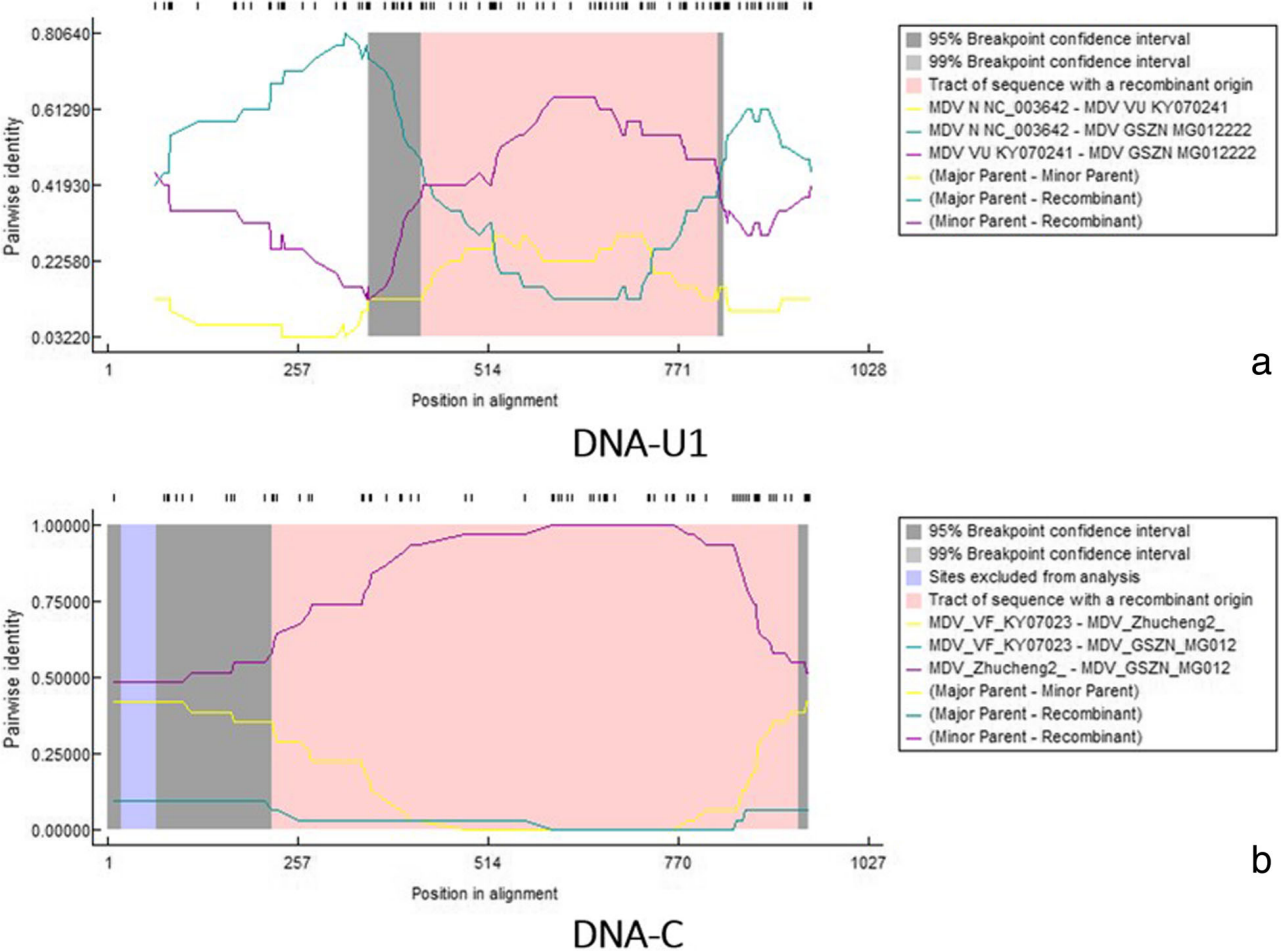
Recombination events depicted in MDV-GSZN isolate: (**a**) DNA-U1; MDV-VU KY07021-like (**b**) DNA-C; MDV Zhuchang2-like. The Dashed line shows the position of nucleotides in the sequence while gray area shows the breakpoint confidence interval

Moreover, both Faba bean necrotic yellows virus (FBNV) isolates grouped together in same clade expect in case of DNA-N where Azerbaijani isolate is placed with Bangladeshi isolates of MDV and Egyptian isolate with two Chinese and one Japanese isolates of MDV that might be the result of possible reassortment reported to be occur in DNA-N of MDV [8]. Recombination was predicted using RDP4.95 [14] to further validate the evolutionary changes. The analysis detected potential recombination events in DNA-C and DNA-U1 segment of MDV-GSZN isolate (Fig. 2) that supported by six out of the seven algorithms: RDP, GENECONV, RecScan, SisScan, MaxChi, Chimera and 3Seq [16–20]. In case of DNA-C , the potential minor parent in Zhucheng 1 (LN890461) with 80% similarity, while the potential major parent is unknown. Whereas, FBNYV (KC979005) is minor parent and BDM1 (LC094161) detected as potential major parent for DNA-U1. The putative multiple parental origin may contribute towards the unusual infectivity behavior of GSZN isolate.

The results of recombination analysis along with phylogenetic study, support the phenomenon of host-shifting and formation of two different groups i.e. legume-infecting and tobacco-infecting in MDV that confirms the ability of this virus to infect plant species out of Fabaceae family. The possible recombination in DNA-C segment of the genome, might play a key role in host specificity i.e. narrow host range of MDV and successful infection in tobacco plant in particular, by initiating viral DNA replication through interacting key regulatory pathways of the host plant cell cycle regulator protein families i.e. pRB and SKP1 [21]. To our knowledge, this is the first full genome analysis of MDV infecting *N. tabacum* in natural field conditions. However, still a comprehensive study is required to further clarify the host shifting dilemma of this virus.

## Additional files

Additional file 1: **Figure S1.** Agarose gel (1.5%) showing the results of restriction enzymes used for digesting RCA products. (left to right) Lane M: 5 kb DNA marker, 1–4: no band, 5: XbaI, 6: 5 kb DNA marker, 7: SamI, 8: SalI, 9: no band, 10–11: HindIII, 12:PstI, 13–14: SacI (no band), 15:BamHI, 16: EcoRI. (PDF 244 kb)

Additional file 2: **Figure S2.** Agarose gel (1%) showing the results of the PCR detection assay of MDV-CP in selected samples of insect transmission experiment. Lane M: DL2,000 bp DNA Marker (Takara); lane C represents the control. (PDF 199 kb)

## Abbreviations

aa: Amino acid
Clink: Cell-cycle link protein
CP: Capsid protein
cssDNA: Circular single-stranded DNA
GSZN: Name of virus isolate used in current study
LAMP: Loop-mediated isothermal amplification
MDV: Milk vetch dwarf virus
MP: Movement protein
M-Rep: Master replication protein
NSP: Nuclear-shuttle protein
nt: Nucleotide
RCA: Rolling circle amplification
