# Supplementary material for: Full genome sequence analysis and putative host-shifting of Milk vetch dwarf virus infecting tobacco (Nicotiana tabacum) in China
Source: Virol J. 2019 Mar 27;16:38. doi: 10.1186/s12985-019-1129-5 (PMC6437909; doi:10.1186/s12985-019-1129-5)
Supplement: Supplementary file 1 — Figure S1. Agarose gel (1.5%) showing the results of restriction enzymes used for digesting RCA products. (left to right) Lane M: 5 kb DNA marker, 1–4: no band, 5: XbaI, 6: 5 kb DNA marker, 7: SamI, 8: SalI, 9: no band, 10–11: HindIII, 12:PstI, 13–14: SacI (no band), 15:BamHI, 16: EcoRI. (PDF 244 kb) [file 12985_2019_1129_MOESM1_ESM.pdf]

Full genome sequence analysis and putative host-shifting of *Milk vetch dwarf virus* infecting tobacco (*Nicotiana tabacum*) in China

Ali Kamran[1, 2, !, #], Han Hou[1, !], Yi Xie[1], Cunxiao Zhao[3], Xiaomin Wei[3], Chaoqun Zhang[4], Xiangwen Yu[5], Fenglong Wang[1, *], Jinguang Yang[1, *]

[1] Key Laboratory of Tobacco Pest Monitoring Controlling & Integrated Management, Tobacco Research Institute of Chinese Academy of Agricultural Sciences, Qingdao 266101, China

[2] Graduate School of Chinese Academy of Agricultural Sciences, Beijing 100081, China

[3] Qingyang Tobacco Company, Gansu Tobacco Cooperation, Xifeng 745000, China

[4] Jiangxi Tobacco Science Institute, Nanchang 330025, China

[5] Sichuan Tobacco Science Institute, Chengdu 610000, China

* Correspondence: yangjinguang@caas.cn, wangfenglong@caas.cn,+86-532-88703236

[!] Authors contributed equally to this work

[#] ORCID: 0000-0002-3651-9395

Name of the Journal: Virology Journal

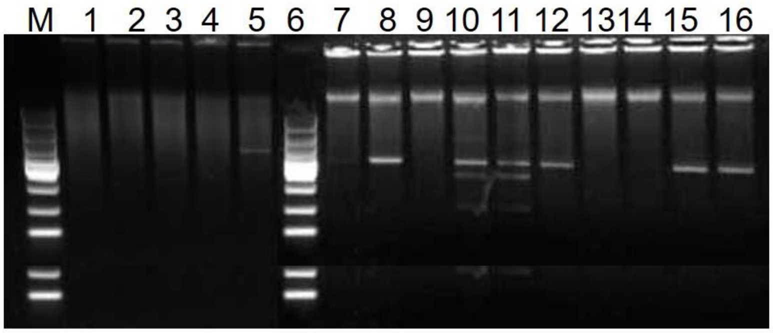

Additional file 1: Figure S1. Agarose gel (1.5%) showing the results of restriction enzymes used for digesting RCA products. (left to right)
Lane M: 5kb DNA marker, 1-4: no band , 5: XbaI, 6: 5kb DNA marker, 7: SamI, 8: SalI, 9: no band, 10-11: HindIII, 12:PstI,13-14: SacI (no band), 15: BamHI, 16: EcoRI
