# Supplementary material for: Full genome sequence analysis and putative host-shifting of Milk vetch dwarf virus infecting tobacco (Nicotiana tabacum) in China
Source: Virol J. 2019 Mar 27;16:38. doi: 10.1186/s12985-019-1129-5 (PMC6437909; doi:10.1186/s12985-019-1129-5)
Supplement: Supplementary file 2 — Figure S2. Agarose gel (1%) showing the results of the PCR detection assay of MDV-CP in selected samples of insect transmission experiment. Lane M: DL2,000 bp DNA Marker (Takara); lane C represents the control. (PDF 199 kb) [file 12985_2019_1129_MOESM2_ESM.pdf]

Full genome sequence analysis and putative host-shifting of *Milk vetch dwarf virus* infecting tobacco (*Nicotiana tabacum*) in China

Ali Kamran[1, 2, !, #], Han Hou[1, !], Yi Xie[1], Cunxiao Zhao[3], Xiaomin Wei[3], Chaoqun Zhang[4], Xiangwen Yu[5], Fenglong Wang[1, *], Jinguang Yang[1, *]

[1] Key Laboratory of Tobacco Pest Monitoring Controlling & Integrated Management, Tobacco Research Institute of Chinese Academy of Agricultural Sciences, Qingdao 266101, China

[2] Graduate School of Chinese Academy of Agricultural Sciences, Beijing 100081, China

[3] Qingyang Tobacco Company, Gansu Tobacco Cooperation, Xifeng 745000, China

[4] Institute of Tobacco Leaf Science, Nanchang 330025, China

[5] Sichuan Tobacco Science Institute, Chengdu 610000, China

* Correspondence: wangfenglong@caas.cn, yangjinguang@caas.cn, +86-532-88703236

[!] Authors contributed equally to this work

[#] ORCID: 0000-0002-3651-9395

Name of the Journal: Virology Journal

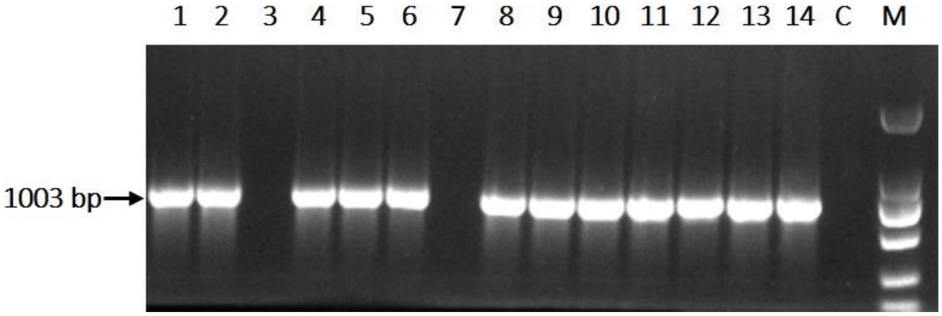

Additional file 2: Figure S2. Agarose gel (1%) showing the results of the PCR detection assay of MDV-CP in selected samples of insect transmission experiment. Lane M: DL2,000 bp DNA Marker (Takara); lane C represents the control
